# Community- and Individual-Level Socioeconomic Status and Breast Cancer Risk: Multilevel Modeling on Cape Cod, Massachusetts

**DOI:** 10.1289/ehp.10818

**Published:** 2008-04-25

**Authors:** Thomas F. Webster, Kate Hoffman, Janice Weinberg, Verónica Vieira, Ann Aschengrau

**Affiliations:** 1 Department of Environmental Health; 2 Department of Biostatistics and; 3 Department of Epidemiology, Boston University School of Public Health, Boston, Massachusetts, USA

**Keywords:** breast cancer, latency, multilevel modeling, socioeconomic status

## Abstract

**Background:**

Previous research demonstrated increased risk of breast cancer associated with higher socioeconomic status (SES) measured at both the individual and community levels. However, little attention has been paid to simultaneously examining both measures.

**Objectives:**

We evaluated the independent influences of individual and community SES on the risk of breast cancer using case–control data. Because our previous work suggests that associations may be stronger after including a latency period, we also assessed the effect of community-level SES assuming a 10-year latency period.

**Methods:**

We obtained individual education for cases and matched controls diagnosed between 1987 and 1993 on Cape Cod, Massachusetts (USA). We acquired community-level SES from census data for 1980 and 1990. Using SES data at diagnosis and 10 years earlier, we constructed models for breast cancer risk using individual-level SES only, community-level SES only, and a multilevel analysis including both. We adjusted models for other individual-level risk factors.

**Results:**

Women with the highest education were at greater risk of developing breast cancer in both 1980 and 1990 [odds ratio (OR) = 1.17 and 1.19, respectively]. Similarly, women living in the highest-SES communities in 1990 had greater risk (OR = 1.30). Results were stronger in the analyses considering a latency period (OR = 1.69). Adjusting for intragroup correlation had little effect on the analyses.

**Conclusions:**

Models including individual- or community-level measures of SES produced associations similar to those observed in previous research. Results for models including both measures are consistent with a contextual effect of SES on risk of breast cancer independent of individual SES.

Over the course of her lifetime, approximately one woman out of every eight will develop invasive breast cancer. Breast cancer remains the most commonly diagnosed non-skin cancer and is the second leading cause of cancer death in women (American Cancer Society 2006). Because of its frequency, the causes of breast cancer have been investigated a great deal at both the individual and community levels. On an individual basis, researchers have determined that characteristics such as age, race, religion, and socioeconomic status (SES) are associated with a woman’s risk of developing breast cancer (Kelsey and Horn-Ross 1993). Rates rise sharply with age and are highest among white women and Jewish women. Women of higher SES/socioeconomic position are also more likely to develop breast cancer. In particular, higher individual educational attainment, as well as higher individual income, are associated with increased risk (Heck and Pamuk 1997; Kelsey and Bernstein 1996).

Overall, community-level SES is also associated with breast cancer incidence. Similar to the results of studies that examine individual SES, higher educational attainment and income as measured at the community level are also associated with higher incidence of breast cancer (Devesa and Diamond 1980; Gorey et al. 1998; Mackillop et al. 2000; Yost et al. 2001).

Although previous research has provided much information about these separate associations, few prior studies examined individual and community SES simultaneously. As a result, it is unclear whether the greater breast cancer incidence in high-SES communities is attributable to a greater number of high-SES women living in an area—that is, the composition of the areas—or to some aspect of high-SES communities that confers a greater risk of being diagnosed to all residents, regardless of their SES, that is, a contextual effect. For example, communities with higher SES may have greater access to screening mammography and diagnose more cases of breast cancer, even if the true incidence rates are otherwise similar.

Community-level variables commonly used in ecologic analyses, such as census data, are the aggregate of individual-level observations. It remains unclear whether health risks observed in ecologic studies are compositional, attributable to the risk in the individual components, or contextual, related to some characteristic of place. Ecologic studies are limited by their inability to control for individual-level confounders. Because individuals group themselves by social constructs, the distribution of confounders is not random and may result in biased group-level effect estimates (Diez-Roux 2001). Examining the contextual determinants of risk requires simultaneously evaluating both community- and individual-level variables. The multilevel approach accounts for confounding by individual-level variables and provides an assessment of the association between community-level measures and individual health independent of individual-level variables (Greenland 2000, 2001). Multilevel models cannot generally be analyzed using standard regression techniques because individuals are nested within communities and residual correlation between members of the same community must be addressed.

To the best of our knowledge, only one prior study has attempted to distinguish the impact of individual and community SES measures on breast cancer incidence (Robert et al. 2004). The results of that study suggest that women living in higher-SES communities in Wisconsin have a slightly greater risk of breast cancer than women living in the lower-SES communities [odds ratio (OR) = 1.2; 95% confidence interval (CI), 1.1–1.4] after controlling for individual risk factors, including education. However, the investigators were able to obtain data only regarding residence at date of diagnosis or reference date to characterize community SES. The researchers were unable to examine SES at prior points in time that are likely more relevant to the development of breast cancer.

We previously studied the spatial epidemiology of breast cancer on Cape Cod, Massachusetts (USA), using case–control data and 40-year residential histories, and analyzed individual-level risk factors for the development of breast cancer (e.g., Vieira et al. 2005). We found that breast cancer had stronger geographic associations when we considered a latency period. In the present analyses, we evaluated the hypothesis that individual- and community-level SES measures independently influence a woman’s risk of breast cancer using Cape Cod case–control data. Because our previous work suggests that associations may be stronger after including a latency period, we also assessed the effect of community-level SES assuming a 10-year latency.

## Materials and Methods

### Individual-level variables

We investigated the association between breast cancer and individual- and community-level measures of SES using data from a population-based case–control study of Cape Cod, Massachusetts (USA) (Aschengrau et al. 2003). The Institutional Review Board of Boston University Medical Center approved the research. Briefly, we recruited eligible cases from the Massachusetts Cancer Registry and included women diagnosed with breast cancer from 1987 through 1993 who were permanent residents of eight Cape Cod towns for at least 6 months before diagnosis. We chose controls to represent the underlying population that gave rise to the cases and frequency-matched them to cases based on date of birth in decades and vital status. Because many of the cases were elderly or deceased, we used three sources of controls: *a*) random digit dialing for living controls < 65 years of age, *b*) Centers for Medicare and Medicaid Services (formerly the Health Care Financing Administration) for the living population 65 or more years of age, and *c*) death certificates for controls who had died from 1983 onward. We assigned an index year to all controls based on the diagnosis year of the matched cases. Interviews collected information on numerous established and hypothesized individual-level risk factors, including age, race, height, weight, physical activity, alcohol use, educational attainment, personal and family history of breast cancer (in a mother, sister, or daughter), menstrual and reproductive history, history of mammography, oral contraceptive use, pharmaceutical hormone use, and exposure to ionizing radiation. We also collected a 40-year residential history up to the time of diagnosis or index year.

Because we did not include income data in the original questionnaire, we chose education as the individual-level measure of SES. Although education may be an imperfect measure of SES, it is thought to be a relatively stable indicator because it is generally set early in adulthood. Using a stable measure of individual SES may be advantageous in this population because about half of the study participants were older than retirement age, a point where more variable measures such as income may change rapidly. A single measure of income is less likely than a single measure of education attainment to capture lifetime average financial resources (Heck and Pamuk 1997). Because this research attempts to capture the impact of community-level SES at different time points independent of individual-level SES, using a consistent measure of individual SES may be preferable. Participants reported the highest level of education completed. We divided educational attainment into three categories: low, representing individuals without high school diplomas; medium, individuals with high school diplomas; and high, individuals with at least some postsecondary education.

### Community-level variables

We linked the addresses of study participants to census data from 1980 and 1990. The 1990 data measured community-level SES for the residence at approximately the time of diagnosis or index year; 1980 data measured community-level SES for the residence 10 years previously. We obtained the 1990 data from the U.S. Census Bureau at the census-block-group level (*n* = 141) and at the larger census-tract level (*n* = 34) (U.S. Census Bureau 2006). We purchased community-level data from GeoLytics (East Brunswick, NJ) at the enumeration-district level (*n* = 167) for 1980 because Cape Cod had not yet been tracted. Enumeration districts were similar in size to the 1990 census block groups but did not encompass identical areas.

We considered two community-level measures of SES for each year: percentage of population with incomes below the U.S. poverty line (Krieger et al. 2002, 2003), and the SES composite index previously used by Robert et al. (2004). We broke the percentage of adults with incomes below the U.S. poverty line into three categories based on the 20th and 80th percentiles of the distribution of control women for each analysis. The SES composite index includes median family income, percentage of adults in poverty, percentage of unemployment, and percentage of individuals ≥ 25 years of age who are college graduates.

We divided each category into quintiles based on the distribution of the control women in a particular analysis and assigned a score from 1 to 5. We reverse-coded the percentage of adults in poverty and the percentage of unemployment so that larger values represented areas with higher SES. We then summed the four category scores and divided the composite values again into quintiles. In the composite index, possible SES scores range from 1, representing a low SES, to 5, representing high SES relative to other areas.

Previous research suggests that the association between community-level SES and breast cancer may be confounded by urbanicity (Prehn and West 1998; Robert et al. 2004). We calculated population densities for each block group or enumeration district. We assigned urban, suburban, and rural classifications based on the U.S. Census Bureau classification (U.S. Census Bureau 2002).

### Statistical analysis

Using census data on SES from the time of diagnosis and 10 years earlier, we constructed several models for the risk of breast cancer: *a*) individual-level SES only, *b*) community-level SES only, and *c*) both individual- and community-level SES. We adjusted all models for age, race, body mass index (BMI), alcohol use, personal and family history of breast cancer (in a mother, sister, or daughter), menstrual history, reproductive history (no children, age at first birth below or above 30 years), history of mammography (ever/never), oral contraceptive use, pharmaceutical hormone use, and exposure to ionizing radiation. For multilevel (hierarchical) models, we used generalized estimating equations (GEEs) with a logit link function while assuming a compound symmetric correlation structure. The latter allowed us to take into account residual within-group correlation. For nonhierarchical models, we used logistic regression. We report ORs with 95% CIs. Use of generalized linear mixed models gave very similar results. We used GEEs for several reasons, including an interest in population-averaged effects rather than cluster-specific effects.

## Results

**Figure 1:**
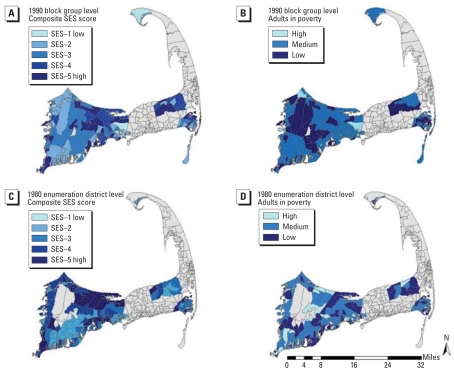
Community-level measures of SES in 1990 and 1980 in areas of Cape Cod, Massachusetts, containing study participants. Darker shades represent areas of comparatively higher SES. Gray areas did not include study participants. Data were not available in 1980 for the Massachusetts Military Reserve, also shown in gray in the middle of the upper Cape. (*A*) Composite SES scores in 1990 by census block group. (*B*) Percentage of adults living in poverty in 1990 by census block group. (*C*) Composite SES scores in 1980 by enumeration district. (*D*) Percentage of adults living in poverty in 1980 by enumeration district.

Figure 1 shows the geographic distribution of the two community-level SES measures in 1980 and 1990. Darker shades represent areas of comparatively higher community-level SES. The composite SES index and the percentage of adults living in poverty produce different estimates of community SES, with areas ranking differently depending on choice of measure.

Table 1 provides information on individual SES and other individual covariates. Individual educational attainment was similar among women in the 1980 and 1990 analyses: Most had at least some postsecondary education, 49.7% and 53.6%, respectively. The study populations were slightly different in 1980 and 1990 due to migration into the area.

Table 2 presents the results of the 1990 analyses. In the model containing only individual-level SES, women with higher educational attainment had an elevated risk of breast cancer compared with women without a high school diploma. The estimated ORs were similar in the high school diploma and postsecondary education groups: 1.30 (95% CI, 0.82–2.07) and 1.19 (95% CI, 0.76–1.88), respectively. In the model examining group-level SES at the block-group level, women in higher-SES communities had an increased risk of developing breast cancer compared with women in the lowest composite SES category (OR = 1.31; 95% CI, 0.86–2.00). The effect of the composite SES variable was not substantially diminished by inclusion of individual-level SES. Adjusting for possible intragroup correlation had little effect on the analysis; the residual intragroup correlation was extremely small. Thus, logistic regression provides results similar to those from analysis with GEE (data not shown). The results of the census-tract–level analyses were similar to the block-group–level analyses, with community-level SES effects generally slightly smaller when measured at the tract level (data not shown). Analyses including urbanicity were quantitatively similar at both the block-group and census-tract levels (data not shown).

Using the other measure of community-level SES, percentage of the adult population with incomes below the U.S. poverty line, the OR estimates at the block-group level were similar to the results using the SES composite index. Compared with women living in communities with the highest percentages of adults with incomes below the poverty line, women in middle- and low-poverty communities had an increased risk of developing breast cancer, OR = 1.16 (95% CI, 0.81–1.66) and OR = 1.30 (95% CI, 0.85–1.99), respectively. These estimates were not substantially altered by the inclusion of the individual-level SES measures. The results at the census-tract level were similar, with both low and medium poverty having increased odds of developing breast cancer, OR = 1.28 (95% CI, 0.84–1.95) and OR = 1.07 (95% CI, 0.75–1.52), respectively.

Table 3 presents results for 1980, examining the effect of community-level SES 10 years before diagnosis or index year. OR estimates for individual-level SES were quite similar to those observed in 1990. Women with a high school diploma had an estimated OR of 1.32 compared with women with no high school diploma, and women with some postsecondary education had an OR of 1.26. Using the composite SES index, women living in the highest-SES communities had a significantly greater risk of developing breast cancer, OR = 1.80 (95% CI, 1.03–3.14), compared with women living in the lowest-SES communities. The effect of the community-level SES was stronger than in the 1990 analysis and was not substantially diminished by inclusion of individual-level SES. Again, adjusting for intragroup correlation had little effect on the analysis. Because the study populations were not identical in 1980 and 1990 (due to migration), we performed another analysis restricted to people who were living in the study area in both years. The results were qualitatively similar.

The 1980 analysis using percentage of adults with incomes below the poverty line as the measure of community-level SES produced effect estimates near the null. The percentage of adults with incomes below the poverty line was more variable in 1980 than in 1990, producing quite different cut points for the 20th and 80th percentiles. To determine whether these differences may have contributed to the change in effect size between 1990 and 1980, we conducted a second analysis applying the 1990 cut points to the 1980 data. Again, the results were near the null (data not shown).

Because of missing covariate data, we did not use approximately 18% and 15% of the observations in the 1980 and 1990 analyses, respectively. Family history, hormone use, and oral contraceptive use had the largest percentage of missing values, between 5% and 8% each. To determine whether our results were sensitive to missing data, we performed multiple imputation using IVEware (Raghunathan et al. 2006). The results for the individual-level SES measure of education were quite similar after imputation (data not shown). The results for the composite SES index were somewhat dampened after imputation, but the 1980 results continued to be stronger than the 1990 results (data not shown).

## Discussion

The individual-level results suggest that higher educational attainment is associated with increased breast cancer risk. Although imprecise (with wide CIs encompassing 1), they are consistent with the magnitude of risk observed in other individual-level analyses using education as a measure of SES (Heck and Pamuk 1997; Robert et al. 2004). The individual-level results were very similar for 1980 and 1990. Although we assessed individual SES only at the time of diagnosis, we believe that it is likely that educational attainment was generally the same in 1980 as it was in 1990, given the advanced age of most women in the study.

Community-level effects were also similar to those observed in other research (Devesa and Diamond 1980; Gorey et al. 1998; Mackillop et al. 2000; Yost et al. 2001). The magnitude of the effect for current community-level SES appears similar to that found by Robert et al. (2004) using the same composite index.

The effect of community-level SES near diagnosis (1990) was similar using both the composite SES measure and the percentage of adults living in poverty. Measured 10 years earlier (1980), the effect of the poverty variable was much smaller than the composite SES index. The difference may be attributable to the inclusion of three additional community-level variables in the composite measure: family income, unemployment, and education. Alternatively, the meaning of poverty may have changed on Cape Cod between 1980 and 1990. The poverty line is set nationally and does not account for regional differences in the cost of living. The 1980 poverty variable may not have captured important differences between the lowest-and highest-SES groups on Cape Cod.

Neither individual- nor community-level effects of SES changed substantially when adjusting for the other. This implies that the SES measures on the two levels are not highly correlated. We constructed the community-level SES measures from census data for all adults (the composite index includes poverty, family income, unemployment, and education), whereas the individual-level measure examines highest education achieved in a predominantly older female group. Our results are consistent with a contextual effect of SES on the risk of breast cancer independent of individual-level SES. They suggest there is something about living in high-SES communities that results in an increased risk of breast cancer independent of individual-level SES. Alternatively, the community-level SES may be capturing an unmeasured aspect of individual-level SES. Individual educational attainment alone may not sufficiently characterize the association between SES and breast cancer; the community-level variables may operate as proxies for residual individual-level information. Our results may be of interest to both social epidemiologists investigating contextual effects and environmental epidemiologists searching for better ways to adjust for SES.

The effect of the composite index of community-level SES was somewhat stronger when measured 10 years before diagnosis, suggesting the importance of latency for the contextual effect. This phenomenon had not been previously studied for breast cancer and only rarely examined for other health outcomes. The investigation and meaning of latency in multilevel models deserve more attention; studies that do not consider latency may underestimate contextual effects.

Although our results suggest an increased risk of breast cancer in higher-SES communities independent of individual SES, the mechanism of action remains unclear. It has been hypothesized that access to mammography is a possible explanation for the greater risk observed in high-SES communities (Katz et al. 2000; Robert et al. 2004). Adjusting for mammography somewhat reduced the effects of individual- and group-level SES on breast cancer risk in our population, but left the basic pattern the same (data not shown). However, we could only control for women ever having had a mammogram or not; more detailed information about timing and frequency of mammograms may make a larger difference. The mechanism for contextual effects of community SES measured 10 years before diagnosis is unknown, but could possibly be related to “social exposures” earlier in life. Community SES could potentially also be related to geographically defined environmental exposures.

Our study has several limitations. Although we have 40-year residential histories and have shown elsewhere that spatial variation in breast cancer risk on Cape Cod increased assuming 20 years of latency (Vieira et al. 2005), we were unable to obtain census data for earlier than 1980 at a small enough scale to perform the multilevel analyses. The size of the census districts were similar in 1980 (enumeration districts) and 1990 (block groups), but the boundaries differed. Determination of the most appropriate geographic scale and measures for community-based variables remains a challenge and adds to the potential for misclassification of community-level SES. Our control selection procedure may not fully capture the experience of people who were permanent residents in the study area at the time cases were diagnosed but moved out of the area before we selected controls. On the other hand, we were able to exclude controls who had not moved to the area at the time of case diagnosis.

## Conclusions

Women living in higher-SES communities had an increased risk of developing breast cancer independent of their own SES. This suggests that a characteristic of high-SES communities may increase the risk of breast cancer apart from individual-level risk factors. Alternatively, the community-level measure may be capturing an unmeasured aspect of individual-level SES. Latency assumptions should also be considered when investigating community-level SES.

## Figures and Tables

**Table 1:** Individual-level SES and other characteristics of women in the 1980 and 1990 analyses [*n* (%)].

Characteristic	1980	1990	Characteristic	1980	1990
Education			Alcohol use		
Less than high school	104 (12.8)	141 (11.2)	Never	134 (16.5)	180 (14.2)
High school diploma	293 (36.1)	432 (34.2)	Once per month	268 (33.1)	390 (30.9)
Some postsecondary	403 (49.7)	678 (53.6)	Few times per month	146 (18.2)	259 (20.5)
Missing	11 (1.4)	13 (1.0)	Few times per week	161 (19.9)	268 (21.2)
Age (years) at diagnosis or index years			Most days	101 (12.5)	165 (13.1)
1–49	132 (16.3)	209 (16.5)	Missing	1 (0.1)	2 (0.0)
50–59	94 (11.6)	163 (12.9)	BMI		
60–69	223 (27.5)	389 (30.8)	Low (< 21)	170 (21.0)	251 (19.9)
70–79	245 (30.2)	345 (27.3)	Medium (21–25)	450 (55.5)	722 (57.1)
≥ 80 years	117 (14.4)	158 (12.5)	High (≥ 26)	171 (21.1)	263 (20.8)
Vital status			Missing	20 (2.5)	28 (2.2)
Deceased	224 (27.6)	334 (26.4)	Hormone use		
Living	587 (72.4)	930 (73.6)	Yes	180 (22.2)	281 (22.2)
Family history of breast cancer			No	570 (70.3)	899 (71.1)
Yes	164 (20.2)	261 (20.6)	Missing	61 (7.5)	84 (6.6)
No	585 (72.1)	926 (73.3)	Oral contraceptive use		
Missing	62 (7.6)	67 (5.3)	Yes	189 (23.3)	303 (24.0)
Personal history of breast cancer			No	569 (70.2)	892 (70.6)
Yes	37 (4.6)	63 (5.0)	Missing	53 (6.5)	69 (5.4)
No	769 (95.4)	1,194 (94.5)	Menopause		
Missing	5 (7.6)	7 (0.5)	Yes	705 (86.9)	1,097 (86.8)
Age at first birth			No	106 (13.7)	167 (13.2)
No children	171 (21.1)	279 (22.1)	Ionizing radiation exposure		
< 30 years	518 (63.9)	796 (63.0)	Yes	45 (5.5)	70 (5.5)
≥ 30 years	108 (13.3)	72 (5.7)	No	746 (92.0)	1,161 (91.9)
Missing	14 (1.7)	17 (1.3)	Missing	20 (2.5)	33 (2.6)
Race			Mammography		
White	780 (96.2)	1,228 (97.2)	Ever	87 (10.7)	1,071 (84.7)
Other	31 (3.8)	36 (2.8)	Never	673 (83.0)	123 (9.7)
			Missing	51 (6.3)	70 (5.5)

**Table 2:** ORs (95% CI) for the association of breast cancer incidence and individual- and community-level SES variables at the block-group level in 1990^a^ (548 cases and 490 controls).

		Composite SES measure		Adults in poverty measure	
	Individual	Group	Multilevel	Group	Multilevel
Individual-level education					
Low	1.00 (reference)		1.00 (reference)		1.00 (reference)
Medium	1.30 (0.82–2.07)		1.32 (0.84–2.06)		1.28 (0.83–1.98)
High	1.19 (0.76–1.88)		1.19 (0.74–1.94)		1.17 (0.75–1.83)
Community-level SES^b^					
1 (low)		1.00 (reference)	1.00 (reference)		
2		1.34 (0.83–2.15)	1.34 (0.80–2.24)		
3		1.21 (0.78–1.88)	1.22 (0.82–1.81)		
4		1.14 (0.72–1.82)	1.13 (0.72–1.77)		
5 (high)		1.31 (0.86–2.00)	1.30 (0.86–1.96)		
Community-level poverty					
High				1.00 (reference)	1.00 (reference)
Medium				1.16 (0.81–1.66)	1.13 (0.83–1.54)
Low				1.30 (0.85–1.99)	1.27 (0.85–1.92)

a The minimum number of cases or controls in any individual or group SES category was 54.

b Composite community SES index at the block-group level (Robert et al. 2004).

**Table 3:** ORs (95% CI) for the association of breast cancer incidence and individual- and community-level SES variables at the enumeration-district level in 1980^a^ (349 cases, 298 controls).

		Composite SES measure		Adults in poverty measure	
	Individual	Group	Multilevel	Group	Multilevel
Individual-level education					
Low	1.00 (reference)		1.00 (reference)		1.00 (reference)
Medium	1.32 (0.76–2.29)		1.28 (0.75–2.19)		1.36 (0.82–2.23)
High	1.26 (0.72–2.19)		1.17 (0.70–1.98)		1.31 (0.78–2.19)
Community-level SES^b^					
1 (low)		1.00 (reference)	1.00 (reference)		
2		1.14 (0.63–2.07)	1.12 (0.64–1.96)		
3		1.23 (0.72–2.11)	1.22 (0.76–1.96)		
4		1.31 (0.75–2.30)	1.28 (0.89–1.84)		
5 (high)		1.80 (1.03–3.14)	1.69 (1.10–2.59)		
Community-level poverty					
High				1.00 (reference)	1.00 (reference)
Medium				1.10 (0.69–1.76)	1.07 (0.70–1.64)
Low				0.98 (0.59–1.64)	0.94 (0.59–1.48)

a The minimum number of cases or controls in any individual or group SES category was 39.

b Composite community SES index at the enumeration-district level (Robert et al. 2004).
